# Effect of auditory interventions on pain in premature infants: a systematic review and meta-analysis

**DOI:** 10.1590/1806-9282.20241000

**Published:** 2025-03-17

**Authors:** Elif Doğan, Gülçin Bozkurt, Rukiye Duman

**Affiliations:** 1 Istanbul University-Cerrahpasa, Faculty of Health Sciences, Department of Midwifery – İstanbul, Turkey.

## INTRODUCTION

The endocrine and immune systems of premature infants are more sensitive to chronic problems and stressful events^1^. Premature infants have more difficulties in the process of adaptation to postnatal life than term infants, and they are highly exposed to painful procedures. Premature infants are most frequently exposed to blood sampling (heel lance, intravenous blood sampling, etc.) among painful invasive procedures^2^. Evidence-based guidelines recommend multidimensional approaches including environmental, pharmacologic, and non-pharmacologic methods in the management of neonatal pain. Pharmacologic methods effectively reduce pain. However, due to the side effects of drugs, it is recommended to primarily use non-pharmacological methods, such as non-nutritive sucking, kangaroo care, breastfeeding, and musical interventions^3^.

Music or some sounds used in pain management in infants show analgesic effects by providing sensory stimulation^4^. It has been reported that various auditory interventions reduce pain in premature infants by blocking irregular noise in the external environment^5^. It was determined that pain was significantly reduced and relieved in newborns who were played music^6^ or mother's voice^7^ during painful procedures^8^. White noise was found to reduce pain, which positively affects physiological parameters (heart rate and respiration) and growth in premature infants^9^.

Although various non-pharmacologic methods have been used in the management of pain in premature newborns, there is a limited number of systematic reviews and meta-analyses examining the effect of auditory interventions used in blood collection procedures on pain levels. Therefore, the aim of this study was to evaluate the effect of auditory pain control methods applied during blood collection procedures on pain levels in premature infants and to provide scientific evidence for the clinical care of newborns.

## METHODS

This study was designed as a systematic review and meta-analysis to evaluate the effect of auditory interventions on pain levels during blood sampling in premature infants.

This systematic review and meta-analysis, which included randomized controlled trials (RCTs), followed the PRISMA checklist^10^ and the Cochrane Handbook for Systematic Reviews of Interventions^11^ for creating the study protocol and writing the article. Before starting the study, it was checked whether this topic was among the previously completed or ongoing studies (registration number: CRD42024551155) from the International Prospective Register of Systematic Reviews (PROSPERO) system.

Literature database: The publications meeting the inclusion criteria were searched between May and July 2024. The screening of included articles, selection of articles, data collection, and quality assessment were performed independently by two researchers (ED and RD), and all stages were controlled by another researcher (GB). In case of any disagreement about the study, a meeting was held with all three researchers to discuss the disagreements and reach a consensus. A search was done in the PubMed, Embase, Web of Science, and Cochrane Library databases with the following keywords: "pain" AND "premature" OR "preterm" AND "auditory intervention" ("music" OR "sound" OR "voice" OR "white noise" OR "lullaby") AND "premature infant pain profile" AND "painful procedures."

Inclusion criteria: The studies to be included in the systematic review were identified according to the following criteria (PICOS):

- Study group (P): Premature infants from whom blood was drawn,
- Intervention (I): Auditory pain management interventions,
- Comparison (C): Premature infants without auditory pain management,
- Outcomes (O): Change in Premature Infant Pain Profile (PIPP) scale score,
- Study design (S): Included RCTs.

Exclusion criteria: The exclusion criteria were as follows: (1) non-RCT studies; (2) studies whose participants were not all premature infants; (3) studies with unclear methods;(4) studies that were not suitable for analysis and had missing data; (5) studies whose full text could not be accessed, and (6) studies that were not published in English.

Quality assessment of the included studies: We used the Cochrane Risk-of-Bias Tool for Randomized Trials V2 (RoB 2) to assess the methodological quality and risk of bias of the included studies. The following criteria were used to assess the overall risk of bias using RoB 2^12^. There are five risk-of-bias domains in this assessment tool. Each risk-of-bias domain is assessed as "low risk," "uncertain risk," and "high risk." The evaluation process was conducted independently by two researchers.

Statistical methods: The meta-analysis was performed with Review Manager 5.3 (the Nordic Cochrane Centre, Copenhagen, Denmark). Effect size was calculated by the meta-analysis for each outcome variable reported in more than one study. The mean difference (MD) was calculated for continuous variables. Heterogeneity between studies was assessed using Cochran's Q test and Higgins’ I^2^. Accordingly, random effect results were considered when I^2^>50%, and fixed effect results were considered when I^2^ was smaller. All tests performed were of two-way, and p<0.05 was considered statistically significant.

## RESULTS

**Figure 1:**
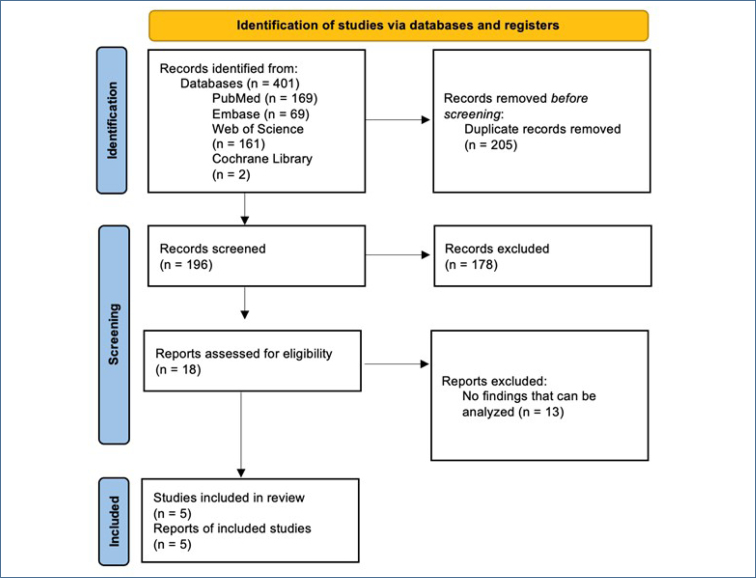
PRISMA flowchart of the screening process.

A total of 196 articles were accessed by searching the databases. The literature search was performed in accordance with the PRISMA checklist, and the flowchart is shown in Figure 1 (Page et al.^10^). A total of five RCTs were included in this study^13–17^.

### The characteristics and quality of the included randomized controlled trials

A total of 629 premature infants from five RCTs were included in this study. Auditory interventions and routine care were applied to 267 premature infants during blood sampling. On comparing the effectiveness of auditory interventions, breast milk and incubator cover were compared in one study, oral sugar solutions in two studies, and kangaroo care in one study. The characteristics of the included RCTs are given in Table 1.

**Table 1:** Characteristics of included studies.

Study	Sample size	Age	Procedure	Auditory intervention	Outcome
Alemdar^13^, Turkey	Mother's voice (n=30) Breast milk (n=30) Incubator cover (n=31) Control (n=32)	30–34 gestational weeks	Periferic cannulation	Mother's voice was played at 45 dB from 15 min before to 15 min after the procedure.	Primary outcomes: PIPP scores were similar compared to the other groups. Secondary outcomes: Premature Infant Comfort Scale (PICS) scores were similar compared to the other groups.
Barandouzi et al^14^, Iran	Music (n=30) 24% oral sucrose (n=30) Music+24% oral sucrose (n=30) Control (n=30)	32–35 gestational weeks	Heel lance	Braham's lullaby was played at 40-50 dB for 10 min, starting 2 min before the procedure.	Primary outcomes: At 30 s after the painful procedure, the pain scores in the music group and the music+24% oral sucrose group were significantly lower than those in the control group (p=0.009 and p<0.001, respectively). Secondary outcomes: None
Howard et al.^15^, Ireland	Music (n=8) Control (n=8)	<32 gestational weeks	Heel lance	Braham's lullaby was played at 40-50 dB for 5 min, starting with the procedure.	Primary outcomes: PIPP scores were similar compared to the other groups. Secondary outcomes: Cortisol levels were similar compared to the other groups.
Shukla et al.^16^, India	Music (n=49) Kangaroo care (n=50) Music+kangaroo care (n=50) Control (n=51)	28–36 gestational weeks	Heel lance	The music group received instrumental Indian classical flute music at 35-45 dB from mobile devices 2 ft away.	Primary outcomes: Although the music+kangaroo care group had lower PIPP scores than the control group, no significance was found in the music group. Secondary outcomes: None.
Uematsu and Sobue^17^, Japan	Music (n=15) Control (n=10)	28–35 gestational weeks	Heel lance	Braham's lullaby (performed by a Japanese female vocalist) was played at 65-75 dB for 5 min after the procedure.	Primary outcomes: PIPP scores after the heel lance were significantly lower in the music group. Secondary outcomes: After the heel lance, oxygen saturation was reported to be higher at 90 s in the music group.

PICS: Premature Infant Comfort Scale; PIPP: Premature Infant Pain Profile.

### Pain score during and after the procedure

**Figure 2:**
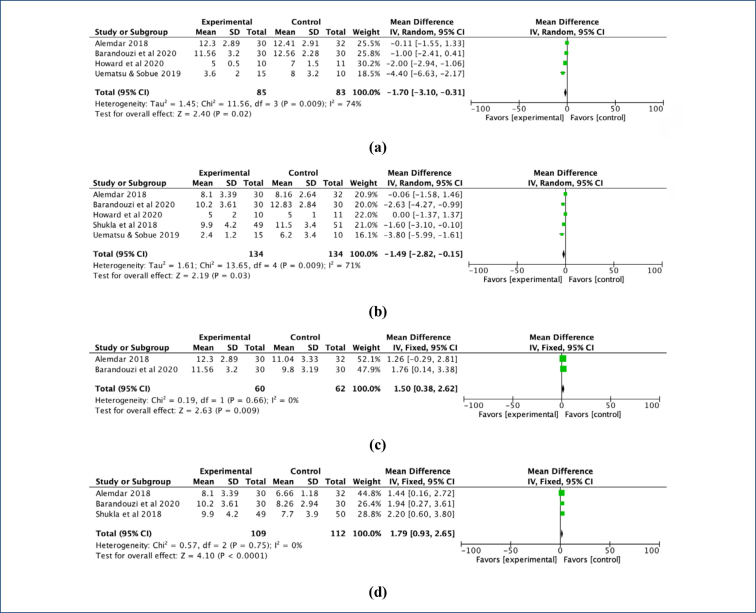
Comparison of PIPP scores: (a) during blood sampling between auditory intervention and control groups, (b) after blood sampling between auditory intervention and control groups, (c) during blood sampling between auditory intervention and other intervention groups, (d) after blood sampling between auditory intervention and other intervention groups. SD: standard deviation, CI: confidence interval.

In all the included studies, PIPP scores were similar in all groups before intervention. From the combined results of these studies, it was observed that PIPP scores were lower in premature infants who received auditory interventions compared to premature infants who received routine care (MD=-1.70, Z=2.40, p=0.02) (Figure 2a). According to the pooled results of PIPP scores obtained after the procedure, lower pain was observed in those who received musical interventions compared to those who received routine care (MD=-1.49, Z=2.19, p=0.03) (Figure 2b).

In three studies, auditory interventions were compared with other interventions (use of breast milk and incubator cover in one study, use of oral sucrose [24%] in one study, and kangaroo care in one study). According to the pooled results of these studies, it was determined that PIPP scores were higher in those who received auditory interventions compared to those who received other interventions (MD=1.50, Z=2.63, p=0.009) (Figure 2c). From the combined results of PIPP scores measured after the procedure, higher pain was observed in those who received auditory interventions compared to those who received other interventions (MD=1.79, Z=4.10, p<0.0001) (Figure 2d).

### Risk of publication bias

A funnel plot was used to evaluate the publication bias of the study. Visual examination showed that the graph was symmetrically distributed and the risk of publication bias was low.

## DISCUSSION

The findings of this study showed that auditory interventions were effective in reducing pain compared to routine care. Auditory interventions can be used as an alternative method to reduce pain in premature infants exposed to painful procedures. Similarly, in another meta-analysis, it was found that maternal voice was effective in reducing pain in newborns^18^. Dur et al. found that white noise and classical music reduced pain scores in retinopathy of prematurity (ROP) examination^19^. In another study, it was determined that the pain scores of newborns exposed to mother's voice while taking heel lance blood decreased^20^. As a result, the findings of the present study are similar to the general literature.

Based on the studies reviewed in the present study, it can be observed that auditory interventions had a positive effect on pain control during blood sampling in premature infants. However, when compared with pain control interventions such as the use of oral sucrose and kangaroo care, it was noted that their effect on pain reduction was low. Oral sucrose is widely used in painful procedures in infants^21^. Although no serious side effects have been reported in the use of oral sucrose, the risks for premature infants are not clear. Although an optimal dose of sucrose has not been defined, the American Academy of Pediatrics (APA) recommends the use of 0.1-1 mL/kg of 24% sucrose. In addition, oral sucrose is not recommended in infants younger than 32 weeks of gestational age^22^.

Kangaroo care is an approach that calms and heals newborns physiologically and behaviorally^23^. Studies show that kangaroo care is effective in relieving pain associated with invasive procedures. Compared to pharmacologic interventions, kangaroo care is easy to implement, is economical, and has low risk^4^. In a meta-analysis including 12 RCTs, it was reported that kangaroo care was advantageous in the stabilization of heart rate in invasive procedures compared to other interventions, but its effectiveness in reducing pain was uncertain^24^. The results of the present study suggest that kangaroo care and the use of oral sucrose solutions are more effective in painful procedures than auditory interventions. However, systematic reviews and meta-analyses including more experimental studies are needed to confirm this effect.

When the methodologic quality of the five included studies was evaluated, it was seen that the overall quality of the studies was good, the risk of bias was low, and there was no publication bias, although there were "some risk concerns."

In this meta-analysis, only premature infants whose blood sampling and pain level were assessed with the PIPP score were included. There are many experimental studies in the literature using pain scales other than PIPP for examining different painful procedures. For a more indepth and broad analysis of this subject, studies reviewing experimental types of studies can be conducted in the future. Additionally, systematic review and meta-analysis studies can be designed to investigate the superiority of different senses over each other by conducting randomized controlled studies examining methods that include different senses in the future.

## CONCLUSION

In the literature, the superiority of the methods used in pain management in infants over each other is controversial. In addition, pain management may vary depending on the condition of the infant. According to our findings, auditory interventions effectively reduce pain during procedures such as heel lance and venous blood collection in premature infants. In addition, no side effects of auditory interventions are reported in the literature. Nevertheless, the effect of auditory interventions compared to other methods is controversial. For example, kangaroo care may also be superior to the use of auditory interventions. However, it should be emphasized that the comparison made in this study was not made as auditory interventions versus olfactory interventions, or auditory interventions versus skin-to-skin touch interventions. In conclusion, auditory interventions are simple, safe, inexpensive, and effective methods. Nevertheless, their combined use with other methods may increase their effectiveness.
